# Frailty using the Clinical Frailty Scale to predict short- and long-term adverse outcomes following emergency laparotomy: meta-analysis

**DOI:** 10.1093/bjsopen/zrae078

**Published:** 2024-08-21

**Authors:** Brittany Park, Zena Alani, Edrick Sulistio, Ahmed W H Barazanchi, Jonathan Koea, Alain Vandal, Andrew G Hill, Andrew D MacCormick

**Affiliations:** Department of Surgery, The University of Auckland, Middlemore Hospital, Auckland, Aotearoa New Zealand; Department of Surgery, The University of Auckland, Middlemore Hospital, Auckland, Aotearoa New Zealand; Department of Surgery, The University of Auckland, Middlemore Hospital, Auckland, Aotearoa New Zealand; Department of Surgery, The University of Auckland, Middlemore Hospital, Auckland, Aotearoa New Zealand; Department of Surgery, The University of Auckland, Middlemore Hospital, Auckland, Aotearoa New Zealand; Department of Statistics, The University of Auckland, Auckland, Aotearoa New Zealand; Department of Surgery, The University of Auckland, Middlemore Hospital, Auckland, Aotearoa New Zealand; Department of Surgery, The University of Auckland, Middlemore Hospital, Auckland, Aotearoa New Zealand

## Abstract

**Background:**

Emergency laparotomy has high morbidity and mortality rates. Frailty assessment remains underutilized in this setting, in part due to time constraints and feasibility. The Clinical Frailty Scale has been identified as the most appropriate tool for frailty assessment in emergency laparotomy patients and is recommended for all older patients undergoing emergency laparotomy. The prognostic impact of measured frailty using the Clinical Frailty Scale on short- and long-term mortality and morbidity rates remains to be determined.

**Methods:**

Observational cohort studies were identified by systematically searching Medline, Embase, Scopus and CENTRAL databases up to February 2024, comparing outcomes following emergency laparotomy for frail and non-frail participants defined according to the Clinical Frailty Scale. The primary outcomes were short- and long-term mortality rates. A random-effects model was created with pooling of effect estimates and a separate narrative synthesis was created. Risk of bias was assessed.

**Results:**

Twelve articles comprising 5704 patients were included. Frailty prevalence was 25% in all patients and 32% in older adults (age ≥55 years). Older patients with frailty had a significantly greater risk of postoperative death (30-day mortality rate OR 3.84, 95% c.i. 2.90 to 5.09, 1-year mortality rate OR 3.03, 95% c.i. 2.17 to 4.23). Meta-regression revealed that variations in cut-off values to define frailty did not significantly affect the association with frailty and 30-day mortality rate. Frailty was associated with higher rates of major complications (OR 1.93, 95% c.i. 1.27 to 2.93) and discharge to an increased level of care.

**Conclusion:**

Frailty is significantly correlated with short- and long-term mortality rates following emergency laparotomy, as well as an adverse morbidity rate and functional outcomes. Identifying frailty using the Clinical Frailty Scale may aid in patient-centred decision-making and implementation of tailored care strategies for these ‘high-risk’ patients, with the aim of reducing adverse outcomes following emergency laparotomy.

## Introduction

Emergency laparotomy (EL) traditionally has high morbidity and mortality rates for older patients^1^. The National Emergency Laparotomy Audit (NELA) in the UK has demonstrated that older age is correlated with increased risk of short-term mortality rates, duration of hospital stays and greater likelihood of discharge to care home accommodation following EL^2^. This cohort is at increased risk of physiological impairment and age-related syndromes such as frailty^2^. The NELA and the recent World Society of Emergency Surgery (WSES) guidelines recommend the need for routine frailty screening for patients aged 65 years and older undergoing EL, to identify those who should receive geriatrician-led multidisciplinary comprehensive geriatric assessment (CGA)^3^.

Many frailty screening tools have been investigated in the preoperative elective setting^4^. The Fried Frailty Phenotype and Edmonton Frailty Scale have been frequently investigated and correlated with postoperative complications and delirium^4^. Further large-scale studies investigating frailty in emergency general surgery populations using the Adjusted Clinical Groups frailty defining diagnosis further demonstrate correlation with long-term mortality rates and institutional discharge^5^. However, there are recognized barriers to implementing the aforementioned tools in the real-time emergency setting. A large number of required variables may be difficult to obtain in a time-constrained setting and some of these variables are hard to attain from the acutely unwell patient (such as gait speed and grip strength). At least partly as a result of this, despite a large body of existing research, frailty assessment remains underutilized in the EL setting^4^.

The Clinical Frailty Scale (CFS) has been recommended by the NELA as the most appropriate tool for frailty assessment in EL patients^2,3^. In the elective setting it was identified as most feasible for implementation before surgery, and strongly associated with death and being discharged to an increased level of care^4,6^. Although the CFS dates back to 2005 and has previously been utilized in medical practice, it has only recently been investigated in the setting of emergency general surgery (EGS)^7^. Developed in a large 5-year prospective study of older Canadian participants in the medical outpatient setting, the CFS is highly correlated with the risk of medium-term death and increased level of care^7^. Initially a 7-point Likert scale, the CFS was updated in 2007 to 9 points, classifying the participant from very fit (scoring the participant a 1), through varying degrees of frailty to terminally ill (scoring the participant a 9)^8^. The CFS is based on clinical assessment and, in theory, may be performed before EL under time constraints, making it attractive for the emergency setting^9^. It has further demonstrated accurate identification of frailty and correlation with death in younger cohorts^8^. The prognostic impact of measuring frailty using the CFS on short- and long-term mortality and morbidity rates in all and older patients undergoing EL remains to be determined. If frailty measured in this way were associated with adverse outcomes following EL, use of the CFS tool in the preoperative setting may provide important additional information to aid in patient-centred decision-making and targeted perioperative bundles of care^10^.

Patients living with frailty may have lower physiologic reserves and, therefore, be at higher risk of death up to 1 year, as well as other postoperative morbidity rate outcomes. The aim of this systematic review and meta-analysis was to determine the impact of frailty, defined according to the CFS, on short- and long-term mortality rates following EL. Secondary aims were to assess morbidity rates and functional outcomes, and compare the CFS with and in addition to other risk assessment tools.

## Methods

The protocol for this systematic review and meta-analysis was registered on PROSPERO (International prospective register of systematic reviews) (CRD 42023475586). It is reported according to the Preferred Reporting Items for Systematic Reviews and Meta-Analyses (PRISMA) and Meta-analysis of Observational Studies Epidemiology (MOOSE) guidelines (*Appendix S1*)^11,12^.

### Eligibility criteria

All studies investigating frailty measured using the CFS in the setting of EL were included. The CFS used a Likert scale ranging from 1 to 7 (2005 to 2007)^7^ or 1 to 9 (after 2007)^6^. Studies that did not use the CFS were excluded. Primary outcomes were short- and long-term mortality rates. Secondary outcomes were major complications (Grade III–V) in accordance with Clavien–Dindo^13^, ICU admission, unplanned reoperation, 30-day readmission, increased level of care and comparison with current risk prediction tools.

### Information sources

The electronic databases Scopus, Medline (OVID), Embase (OVID) and Cochrane Central Register of Controlled Trials were systematically searched for studies published between 1980 and February 2024. The bibliographies of systematic reviews on relevant topics and all included studies were further reviewed for additional eligible studies.

### Search strategy

Boolean operators (‘AND’/‘OR’) were used to combine keywords and Medical Subject Headings (MeSH): ‘frail*’, ‘Clinical Frailty Scale’, ‘CFS’, ‘laparotomy’, ‘colorectal surgery’, ‘general surgery’, ‘abdom* surgery’, ‘abdom* operation’, ‘emergent*’, ‘acute*’, ‘obstruction’, ‘ulcer’, ‘perfor*’. The search strategy applied in the Medline (OVID) database is demonstrated in *Appendix S2*. Studies were included in English only, involving adult participants over the age of 18 years and where full paper access was available.

### Study selection

All original studies assessing patient outcomes for frail *versus* non-frail adults using the CFS following EL were included in this review. Frailty was defined by a variable cut-off value ranging from 4 to 6 on both the 7- and 9-point Likert scale (‘vulnerable’ to ‘moderately frail’). In cases where studies included an identical cohort of patients originating from the same institution and across overlapping intervals of time—these were included if different outcomes were reported on. Studies that did not use the CFS to define frailty were excluded, as well as small case series or reports, systematic/literature reviews, abstracts, editorial letters and studies including patients under the age of 18 years.

### Screening process

Duplicate exclusion was conducted using the methods of Bramer *et al.*^14^, using EndNote X9 (Clarivate, Philadelphia, PA, USA). Rayyan web application for systematic reviews was used by two independent reviewers to screen records^15^. Where required, a senior author was involved for discrepancies over study inclusion. Reference lists of all full-text articles included were screened, as were reference lists for systematic reviews on similar subject matter. The final paper inclusion was agreed upon by consensus.

### Data extraction

Microsoft Office Excel 2020 was used to create a data extraction sheet for the included studies. Data extraction was conducted by the first author and checked by the second author. Disagreements were resolved by consensus. Extracted data included: study characteristics (journal, year published, country, number of centres, study interval, study design, operation type, indication for operation, patient characteristics); CFS definition (1–7 or 1–9 Likert scale used, cut-off value for ‘frailty’ and reference); and outcomes (30-day, 6-month and 1-year mortality rates, major complications, ICU admission, unplanned reoperation, 30-day readmission, admission for rehabilitation, increased level of care and comparison of CFS to other risk assessment tools). The corresponding author for each publication was contacted if information was missing or unclear to obtain as much raw data as possible.

### Risk of bias

Quality assessment and risk of bias were performed by the first and second authors for the included studies using the Quality in Prognosis Studies (QUIPS) tool^16^. Agreement on scores was achieved through discussion and input from a senior author.

### Summary measures and synthesis of results

Frailty prevalence and overall incidence rates for all outcomes were reported as pooled prevalence and incidence (with associated 95% c.i.) using R version 3.6.3 (R Foundation for Statistical Computing, Vienna, Austria)^17^. Outcomes reported by individual studies or by individual increasing frailty scores were reported by narrative synthesis, including variables adjusted for and whether reporting for logistic regression analysis was for crude outcomes, adjusted outcomes or both. Cochrane Review Manager version 5.4 was used for meta-analysis and heterogeneity assessment of raw values. Dichotomous outcomes (where a defined cut-off value to define frailty was established) for incidence of events for death, postoperative complications, ICU admission, unplanned reoperation and 30-day readmission were reported as pooled odds ratios with 95% confidence intervals using a Mantel–Haenszel method with a random-effects model. A *P* value of <0.05 was considered significant for all tests. *I*^2^ statistics were used to assess heterogeneity, with a score of 25, 50 and 75% representing low, moderate and high heterogeneity. Funnel plots of the incidence of 30-day mortality rates were used to screen for publication bias. Meta-regression was undertaken in R to examine the effect of individual increasing frailty scale numbers and 30-day mortality rates.

### Subgroup and sensitivity analysis

In cases of significant concern for bias based on the QUIPS risk-of-bias assessment, additional analysis was performed following exclusion of relevant studies and results were reported. A subgroup analysis of older patients (age ≥ 55 years) and 30-day mortality rates following EL was reported separately.

### Transforming and estimating missing data

Studies reporting on postoperative duration of stay in median and interquartile range (i.q.r.) were converted into mean and standard deviation (s.d.) using the statistical method of Luo *et al*^18^.

## Results

### Search results

**Fig. 1:**
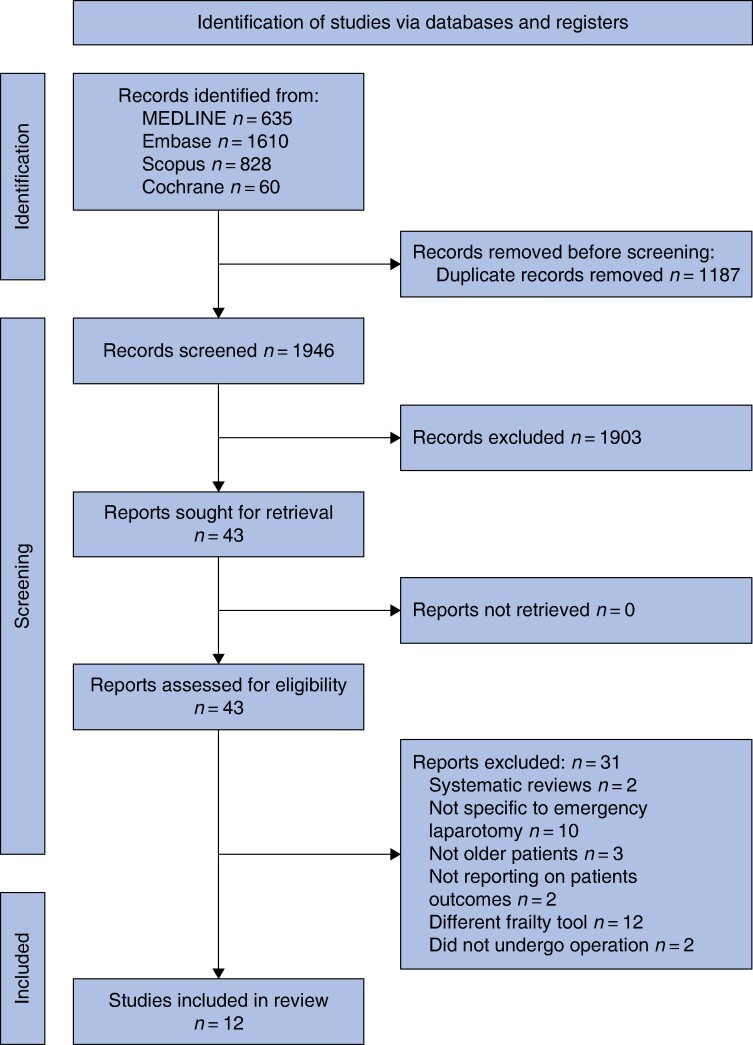
Preferred Reporting Items for Systematic Reviews and Meta-Analyses (PRISMA) diagram for selection of included studies

The initial search identified 3133 records, of which 12 articles on 10 studies were included (*Fig. 1*)^19–30^. Four of these papers reported on identical patient cohorts but different outcomes (Carter and Palmar from the Emergency Laparotomy and Frailty (ELF) study, Ramsay *et al.* from the Emergency Laparoscopic and Laparotomy Scottish Audit (ELLSA) study), hence they were included in the review but equated for frailty prevalence^20,25–27^.

### Study characteristics

Study characteristics are presented in *Table 1*. All studies were published in the last 4 years and examined patients undergoing EL. Most studies were conducted at one centre (*n* = 7). One study was conducted across four centres in New Zealand^28^, the ELLSA study included 18 centres, and the ELF study included 49 centres in the UK respectively^20,25–27^. All studies apart from two included EL as well as major laparoscopic cases in keeping with the NELA definition of EL^21,22^.

**Table 1:** Study characteristics

Study	Country	Number of centres	Study interval	Patient selection	Operation type	Operative indication
Alder *et al.* 2021^19^	UK	1	July 2015–July 2016	Inclusion: all patients over the age of 70 years who underwent emergency laparotomy Exclusion: not stated	Emergency laparotomy and major laparoscopic cases	Not further specified
Carter *et al*. 2020^20^	UK	49	20 March–19 June 2017	Inclusion: if undergoing an expedited, urgent or emergency surgical abdominal procedure for gastrointestinal pathology (laparoscopic or open) and/or return to theatre for any major postoperative complication/dehiscence Exclusion: not stated	Emergency laparotomy and major laparoscopy cases	Gastrointestinal pathology or return to theatre for complication
Ethiraj *et al*. 2022^21^	India	1	March 2021–December 2021	Inclusion: > 60 years undergoing emergency laparotomy for acute surgical conditions Exclusion: patients lost to follow-up, poly trauma patients, and patients with associated medical emergencies	Emergency laparotomy	Emergency laparotomy for acute surgical conditions (obstruction, perforation, abdominal abscess, ischaemia)
Hajibandeh *et al.* 2024^22^	UK	1	January 2017–January 2022	Inclusion: all patients aged over 18 who underwent emergency laparotomy due to non-traumatic abdominal pathology during the time interval Exclusion: patients who underwent emergency laparotomy due to abdominal trauma	Emergency laparotomy	Small and large bowel obstruction, perforated peptic ulcer, small and colonic bowel perforation, ischaemia, intra-abdominal collection, colitis, anastomotic leak
Isand *et al*. 2023^23^	UK	1	January 2018–June 2021	Inclusion: patients who underwent a major emergency abdominal operation corresponded to the NELA inclusion criteria from the same time interval Exclusion: duplicates or missing critical data, not enough information to calculate P-POSSUM score, or NELA score	Emergency laparotomy and major laparoscopic cases	Major emergency abdominal operation corresponded to the NELA inclusion criteria
Palaniappan *et al*. 2022^25^	UK	18	November 2017–October 2018	Inclusion: patients aged 18 and above; admitted or underwent expedited, urgent, or emergency open laparotomy, laparoscopic abdominal procedures Exclusion: <18 years, diagnostic or elective laparotomy/laparoscopy, emergency hernia repair without bowel resection or division of adhesions, all oesophageal, pancreatic, splenic, hepatobiliary, appendiceal, urological, vascular, organ transplant, trauma, obstetric or gynaecological complications	Emergency laparotomy and major laparoscopic cases	Major procedures limited to the stomach, small intestine, large intestine, rectum, intraperitoneal haematomas and abscesses, incarcerated hernias, substantial abdominal wound dehiscence and returns to theatre for elective general surgery complications
Parmar *et al*. 2021^26^	UK	49	March 2017–June 2017	Inclusion: if undergoing an expedited, urgent or emergency surgical abdominal procedure for gastrointestinal pathology (laparoscopic or open) and/or return to theatre for any major postoperative complication/dehiscence Exclusion: *<*18; elective; diagnostic where no subsequent procedure is performed; appendicectomy; cholecystectomy; non-elective hernia repair without bowel resection or division of adhesions; minor abdominal wound dehiscence; non-elective formation of a colostomy or ileostomy as laparoscopic; vascular surgery; caesarean section or obstetric laparotomies	Emergency laparotomy and major laparoscopic cases	Gastrointestinal pathology and/or return to theatre for any major postoperative complication/dehiscence
Ramsay *et al*. 2022^27^	UK	17	November 2017–October 2018	Inclusion: patients undergoing emergency laparotomy Exclusion: previous surgical procedures during same admission	Emergency laparotomy and major laparoscopic cases	Not further specified
Vilches-Moraga *et al*. 2020^28^	UK	1	September 2014–March 2017	Inclusion: aged 75 years or older undergoing emergency laparotomy within the time interval Exclusion: remaining an inpatient > 90 days before the final date of data collection. Patients who had more than one laparotomy on separate admissions were only included for index admission	Emergency laparotomy and major laparoscopic cases	Bowel obstruction/perforation, hernias, peritonitis, ulcers, diverticulitis, ischaemia
Youssef *et al*. 2022^29^	UK	1	December 2018–May 2021	Inclusion: all patients aged 65 years and older who underwent emergency laparotomy Exclusion: patients who had previously undergone surgical procedures during the same admission were excluded	Emergency laparotomy and major laparoscopic cases	Not further specified
Park *et al*. 2024^30^	New Zealand	4	August 2017–September 2022	Inclusion: adult patients aged 55 years or older who were undergoing emergency laparotomy with a recorded CFS Exclusion: < 55, elective laparotomy or laparoscopic procedures, appendicectomy, cholecystectomy, non-elective (urgent or emergent) hernia repair without bowel resection, vascular, gynaecological, urological or transplant procedures	Emergency laparotomy and major laparoscopic cases	Obstruction, perforation, ischaemia, peritonitis, volvulus, haemorrhage, anastomotic leak, relook, abdominal wall abscess, wound dehiscence, fistula, stricture
Mak *et al*. 2024^24^	UK	1	January 2013–December 2016	Inclusion: all patients with colorectal cancer admitted as emergency, not diagnosed via GP referral and elective investigation, and had emergency colorectal cancer operations as primary intervention within the time interval Exclusion: not specified	Emergency laparotomy and major laparoscopic cases	Emergency operations for colorectal cancer including for perforation. Not further specified

NELA, National Emergency Laparotomy Audit; P-POSSUM, Portsmouth-Physiological and Operative Severity Score for the enUmeration of Mortality and Morbidity (POSSUM); CFS, Clinical Frailty Scale; GP, General Practitioner.

### Quality assessment

Results of the quality assessment using the QUIPS tool are presented in *Table S1*. Of the 12 included studies, nine were scored as having a low risk of bias for each of the domains^19,20,22–24,26,28–30^. Palaniappan and Ramsay *et al.* (both reporting on the ELLSA study) received moderate risk of bias for study participation and study attrition domains as 812 participants were missing from prospectively collected data from 18 sites^25,27^. The impact of missing data and characteristics of patients with missing data compared with those analysed was not reported. Ethiraj *et al.* was assessed as having a moderate overall risk of bias. They did not adjust for potential confounding variables and in their statistical reporting did not give confidence intervals or *P* values for odds ratios, resulting in a high risk of bias for each of the respective bias domains^21^.

### Patient characteristics

Emergency laparotomy was performed on 5704 patients. Seven studies focused specifically on older adult cohorts (age ≥ 55 years), the remainder included all adult patients. *Table S2* outlines patient demographics. The majority of patients were aged over 65 years, with an ASA score ≥III.

### Frailty definitions

**Fig. 2:**
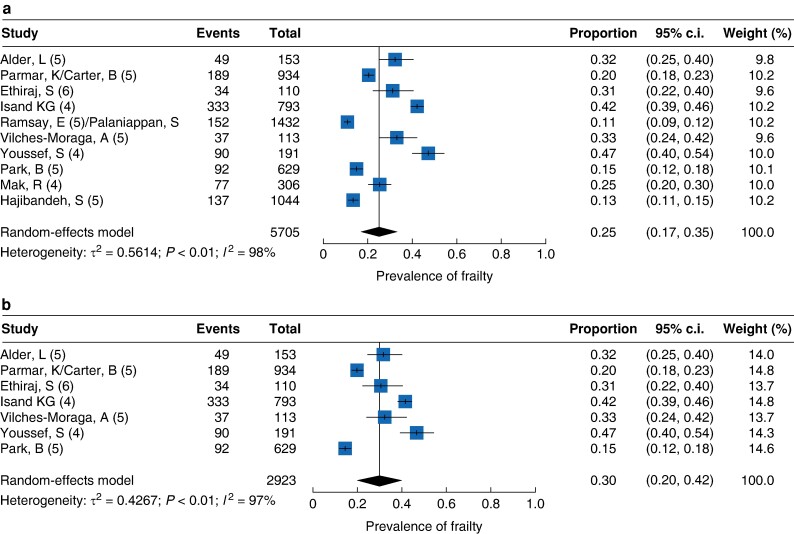
a Frailty overall pooled prevalence. b Frailty prevalence over 55

All studies assessed frailty according to Rockwood’s CFS (*Table 2*). The majority utilized the updated version of the CFS (Likert scale 1–9, *n* = 6)^17,21,23,27–29^, however, five studies used the older scale from 2005 (Likert scale 1–7)^18,20,24–26^. Three studies used a cut-off value of 4 (‘vulnerable’) to define the presence of frailty. Five studies used a cut-off value of 5 (‘mildly frail’) and one study used a cut-off value of 6 (‘moderately frail’). The pooled prevalence of frailty was 25.0% (95% c.i. 0.17 to 0.36, *I*^2^ = 98, *Fig. 2a*). In studies focusing on older patients (aged ≥55 years), pooled frailty prevalence was 32% (95% c.i. 0.20 to 0.48, *I*^2^ = 98, *Fig. 2b*).

**Table 2:** Frailty definitions

Study	Age range (years)	CFS Likert scale	Reference	Cut-off value	Definitions	Frailty prevalence
Alder *et al.* 2021^19^	≥70	1–9	6	≥5	NS	32.0% (49 of 153)
Carter *et al*. 2020/Parmar *et al.* 2021^20,26^	≥65	1–7	5	≥5	1–4 = non-frail, 5–7 = frail	20.2% (189 of 934)
Ethiraj *et al*. 2022^21^	≥60	1–7	5	≥6	1–3 = fit, 4–5 = vulnerable, 6–7 = frail	30.9% (34 of 110)
Hajibandeh *et al*. 2024^22^	All	1–9	6	≥5	NS	13.14% (137 of 1043)
Isand *et al*. 2023^23^	All (and ≥65)	1–9	6	≥4	1–3 = not frail, 4–9 = frail	42.0% (333 of 793—all) 59.4% (244 of 411 – ≥ 65)
Palaniappan *et al*. 2022/Ramsay *et al*. 2022^25,27^	All	1–7	5	≥5	1–3 = not frail, 4 = prefrail, 5 = mildly frail, 6–7 = moderate–severely frail	10.6% (152 of 1432)
Vilches-Moraga *et al*. 2020^28^	≥75	1–9	6	≥5	1–4 = non-frail, 5–9 = frail	32.7% (37 of 113)
Youssef *et al*. 2022^29^	≥65	1–9	6	≥4	1–3 = non-vulnerable, ≥ 4 = frail	47.1% (90 of 191)
Park *et al.* 2024^30^	≥55	1–9	6	≥5	1–4 = non-frail, 5–7 = frail	14.6% (92 of 629)
Mak *et al*. 2024^24^	All	1–9	6	≥4	1–3 = less frail, ≥ 4 = more frail	25.2% (77 of 306)

CFS, Clinical Frailty Scale; NS, not specified.

### Outcome measures

#### Mortality rate

**Fig. 3:**
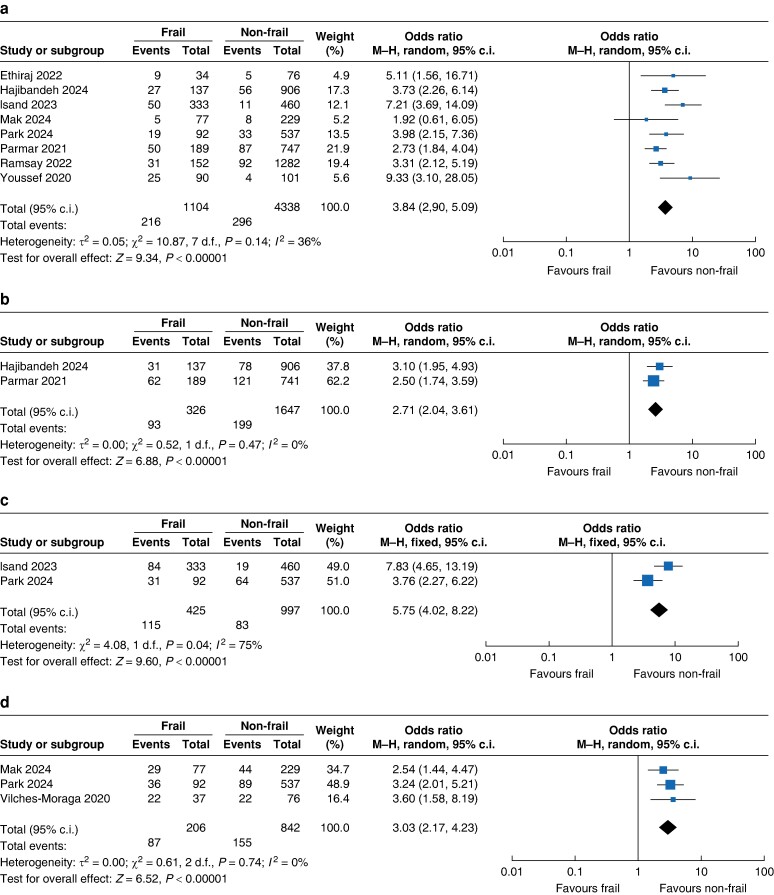

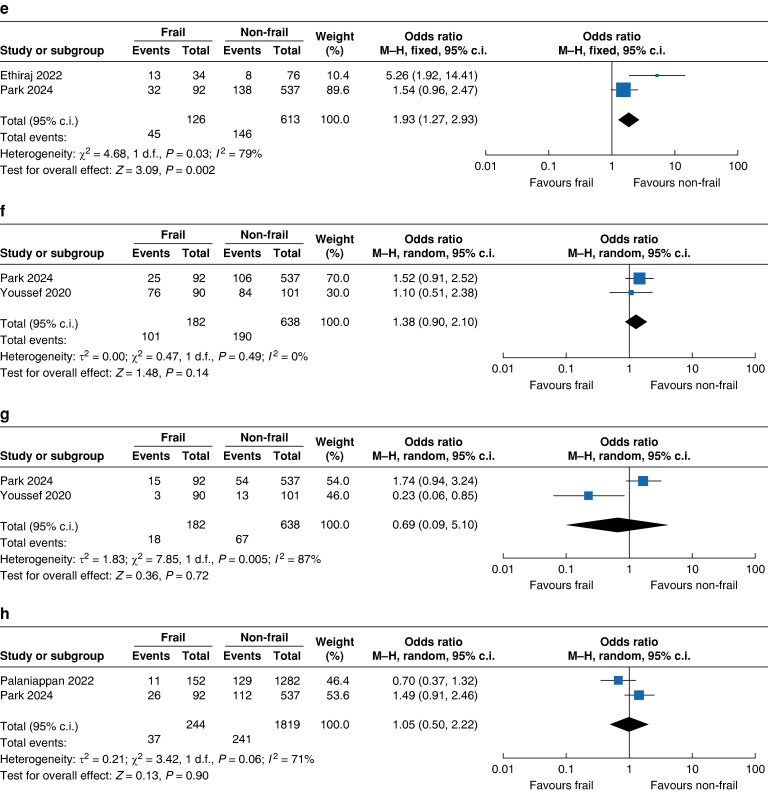
a 30-day mortality rate, b 90-day mortality rate, c 6-month mortality rate, d 1-year mortality rate, e major complications, f ICU admission, g unplanned reoperation, h 30-day readmission

The results illustrate the pooled effect of studies comparing frail and non-frail participants and postoperative short- and long-term mortality rates. The incidences of overall 30-day, 90-day, 6-month and 1-year deaths were 9% (95% c.i. 7 to 13, *I*^2^ = 87%), 14% (95% c.i. 0 to 9.5, *I*^2^ = 97%), 14% (95% c.i. 5 to 33, *I*^2^ = 23%) and 26% (95% c.i. 10 to 54, *I*^2^ = 89) respectively (*Fig. S1a–d*). Frailty defined according to the CFS was associated with a significantly increased incidence of 30-day deaths following EL (*Fig. 3a*, *n* studies = 8, OR 3.84, 95% c.i. 2.90 to 5.09, *P* < 0.001, *I*^2^ = 36%), 90-day mortality rate (*Fig. 3b*, *n* studies = 2, OR 2.71, 95% c.i. 2.04 to 3.61, *P* < 0.001, *I*^2^ = 0%), 6-month mortality rate (*Fig. 3c*, *n* studies = 2, OR 5.90, 95% c.i. 4.13 to 8.42, *P* < 0.001, *I*^2^ = 78%) and 1-year mortality rate (*Fig. 3d*, *n* studies = 3, OR = 3.03, 95% c.i. 2.17 to 4.23, *P* < 0.001, *I*^2^ = 40%) compared with non-frail participants. Heterogeneity ranged from low to high in the included studies.

#### Sensitivity analysis

**Fig. 4:**
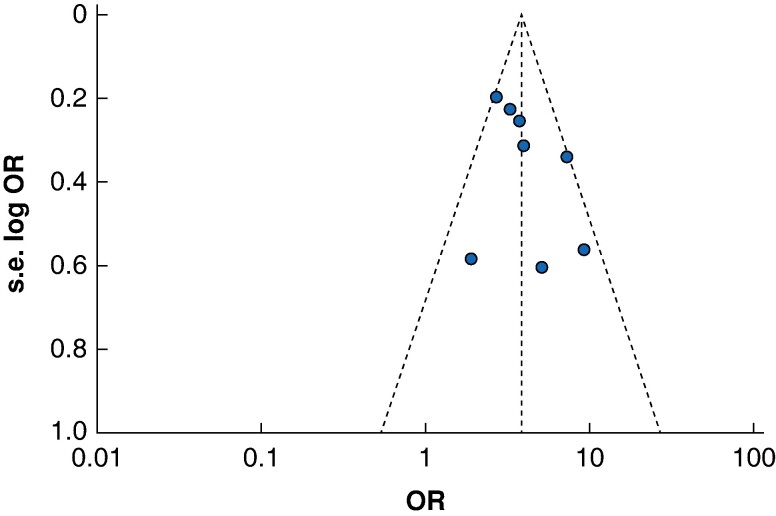
30-day mortality rate funnel plot

After exclusion of one study with a QUIPS score demonstrating a high risk of bias, meta-analysis demonstrated a significant association with frailty and 30-day mortality rates (*n* studies = 7, OR 3.80, 95% c.i. 3.08 to 4.69, *P* < 0.001, *I*^2^ = 43%). Meta-regression analysis for differences in appreciable cut-off values to define frailty did not identify a significant association between cut-off values and 30-day mortality rates across the included studies (R^2^ = 29.12%, *P* = 0.2417). A funnel plot for 30-day mortality rates is available for review in *Fig. 4*, demonstrating no apparent asymmetry for this primary endpoint.

#### Subgroup analysis

Of the four studies that examined frailty in older patients (age ≥55 years), a comparable association with 30-day mortality rate for all patients was demonstrated (*Fig. S2*, *n* studies = 4, OR = 3.94, 95% c.i. 2.45 to 6.34, *P* < 0.001, *I*^2^ = 42%).

#### Mortality rate with increasing frailty

Three studies found that increasing individual CFS scores were correlated with increased risk of 30-day death on unadjusted and adjusted multivariable regression analysis (*Table 3*). Two of these studies adjusted for age and sex, one adjusted for age and NELA score^23,26,27^. One of these studies further demonstrated this association with the 90-day mortality rate^26^.

**Table 3:** Summary of study results

Author	Study design	Retrospective/prospective	Variables adjusted for	Reporting	Significant results
Alder *et al*. 2021^19^	Observational/cohort study	Retrospective	Disease factors, operative approach	Adjusted outcomes	− CFS ≥5 midterm mortality rate Adjusted OR 3.20 (95% c.i. 1.09 to 9.61) (median follow-up 19 months) – ROC curve for CFS ≥5 and midterm death = 0.86 – Following surgery, observed deterioration in CFS from average score 4 to 5
Carter *et al*. 2020^20^	Observational/cohort study	Prospective	Sex, age	Unadjusted and adjusted	− Increasing frailty associated with increase in care level at discharge – OR of increased care on discharge: Unadjusted: CFS 4–5.01 (95% c.i. 2.28 to 10.97); CFS 5–6.04 (95% c.i. 2.69 to 13.58); CFS 6 or 7–6.54 (95% c.i. 2.70 to 15.85) (compared with CFS 1) Adjusted: CFS 4–4.48 (95% c.i. 2.03 to 9.91); CFS 5–5.94 (95% c.i. 2.54 to 13.90); CFS 6 or 7–7.88 (95% c.i. 2.97 to 20.79)
Ethiraj *et al*. 2022^21^	Observational/cohort study	Prospective	–	Unadjusted outcomes	− CFS ≥ 6 associated with 30-day mortality rate Unadjusted OR 4.20 (95% c.i. not presented) – Increased LOS for frail participants Unadjusted OR 2.23 – Increase in postoperative complications Unadjusted OR 3.63
Hajibandeh *et al*. 2024^22^	Observational/cohort study	Retrospective	Age, age ≥ 80 years, ASA status, need for bowel resection, presence of peritoneal contamination, CFS, sarcopenia	Adjusted outcomes	− The CFS was not a predictor of 30-day mortality rate—adjusted OR: 1.10 (95% c.i. 0.88 to 1.38) – Sarcopenia is a stronger predictor of 30-day mortality rate (AUC: 0.87 *versus* 0.70), in-hospital death (AUC: 0.79 *versus* 0.67), and 90-day mortality rate (AUC: 0.79 *versus* 0.67) compared with CFS
Isand *et al*. 2023^23^	Observational/cohort study	Retrospective	NELA RPT predicted mortality rate, age	Adjusted outcomes	− Adding CFS into the P-POSSUM and NELA-RPT models improved both tools in the elderly (AUC increased from 0.775 to 0.846 (*P* < 0.05) and from 0.814 to 0.864 (*P* < 0.05) respectively – Increasing OR of 30-day mortality rate with increasing frailty. Adjusted OR CFS 5–5.0 (95% c.i. 2.2 to 11.7), CFS 6–13.5 (95% c.i. 6.1 to 30.1), CFS 7–34.5(95% c.i. 12.3 to 96.6) (*versus* 1–3)
Mak *et al.* 2024^24^	Observational/cohort study	Retrospective	–	Unadjusted outcomes	− Frail patients (CFS ≥ 4) had a higher mortality rate at 90 days (16.9% *versus* 5.2%, *P* < 0.05), 1 year (37.7% *versus* 19.2%, *P* < 0.05) and 3 years (61.0% *versus* 45.4%, *P* < 0.05)
Palaniappan *et al.* 2022^25^	Observational/cohort study	Prospective	Sex, age	Unadjusted and adjusted	− As CFS increased so did the 30-day mortality rate (2.1% for CFS 1, 25.3% for CFS 6 and 7, *P* < 0.001) and median LOS (10 days for CFS 1, 20 days for CFS 6 and 7, *P* < 0.001) – ROC for the mortality rate was 0.71 (95% c.i. 0.65 to 0.77) for CFS and 0.84 (95% c.i. 0.78 to 0.89) for NELA – Addition of CFS to NELA did not increase ROC value – Readmission rates did not differ significantly at any CFS value compared with a CFS of 1 on both unadjusted and adjusted analysis – Frailty associated with increased duration of hospital stay on unadjusted but not adjusted analysis – Risk of long *versus* short duration of stay: Unadjusted OR: CFS 4–3.08 (95% c.i. 2.08 to 4.56), CFS 5–4.78 (95% c.i. 2.70 to 8.46), CFS 6 and 7–4.48 (95% c.i. 2.52 to 7.97) (compared with CFS 1) Adjusted OR: CFS 4–1.35 (95% c.i. 0.82 to 2.20), CFS 5–1.70 (95% c.i. 0.88 to 3.29), CFS 6 and 7–1.76 (95% c.i. 0.89 to 3.46) (compared with CFS 1)
Parmar *et al*. 2021^26^	Observational/cohort study	Prospective	Sex, age	Unadjusted and adjusted	− Frailty associated with the following outcomes on unadjusted and adjusted analysis: – 90-day mortality rate – Unadjusted OR 90-day mortality: CFS 5 OR 3.12 (95% c.i. 1.24 to 7.99) and CFS 6/7 OR 5.89 (95% c.i. 2.19 to 15.86) (compared with CFS 1) – Adjusted OR 90-day mortality rate: CFS 5–3.18 (95% c.i. 1.24 to 8.14) and CFS 6/7–610 (95% c.i. 2.26 to 16.45) (compared with CFS 1). Similar associations for the 30-day mortality rate – Increased risk of complications: – Unadjusted OR risk of complications: CFS 5–4.42 (95% c.i. 2.11 to 9.24) and CFS 6/7–3.78 (95% c.i. 1.64 to 8.73) – Adjusted OR risk of complications: CFS 5–4.56 (95% c.i. 2.17 to 9.60) and CFS 6/7–3.92 (95% c.i. 1.69 to 9.10) – Increased duration of ICU stay: – Unadjusted OR duration of ICU stay: CFS 5–2.11 (95% c.i. 1.14 to 3.89) and CFS 6/7–4.00 (95% c.i. 2.00 to 7.98) – Adjusted OR duration of ICU stay: CFS 5–2.15 (95% c.i. 1.15 to 3.96) and CFS 6/7–4.18 (95% c.i. 2.11 to 8.03) – Increased OR of postoperative hospital stay: – Unadjusted OR increased postoperative hospital stay: CFS—OR 1.46 (95% c.i. 1.11 to 1.91) and CFS 6/7–1.64 (95% c.i. 1.21 to 2.23) Adjusted OR increased postoperative hospital stay: CFS—OR 1.44 (95% c.i. 1.10 to 1.89) and CFS 6/7–1.62 (95% c.i. 1.19 to 2.20) – 30-day readmission—no significant relationship identified
Park *et al*. 2024^30^	Observational/cohort study	Prospective	Sex, age, ethnicity	Unadjusted and adjusted	− Direct associated with frailty (CFS ≥ 5 *versus* <5) and short- and long-term mortality rates: – 30-day mortality rate Unadjusted RR: 3.0 (95% c.i. 1.8 to 4.9), adjusted RR: 2.6 (95% c.i. 1.5 to 4.3) – 1-year mortality rate Unadjusted RR 2.3 (95% c.i. 1.7 to 3.2), adjusted RR: 2.0 (95% c.i. 1.5 to 2.8) – Increased risk of admission for rehabilitation – Unadjusted RR 3.0 (95% c.i. 2.1 to 4.3), adjusted RR 2.1 (95% c.i. 1.4 to 3.0), – Discharge to an increased level of care Unadjusted RR 3.5 (95% c.i. 1.9 to 6.7), adjusted RR 2.1 (95% c.i. 1.1 to 4.2) – Major complications Unadjusted RR 1.5 (95% c.i. 1.2 to 1.9), adjusted RR 1.5 (95% c.i. 1.1 to 1.9) – Readmission within 30 days Unadjusted RR 1.4 (95% c.i. 1.01 to 2.1), adjusted RR 1.6 (95% c.i. 1.1 to 2.3)
Ramsay *et al*. 2022^27^	Observational/cohort study	Prospective	Sex, age	Adjusted outcomes	− Higher frailty scores correlated with longer process measure timings across all measures of temporal processes of care – Increasing 30-day mortality rate OR with increasing frailty: Adjusted OR 30-day mortality rate: CFS 4 OR 2.75 (95% c.i. 1.68 to 4.50), CFS 5 OR 2.24 (95% c.i. 1.12 to 4.46), CFS 6–7 OR 4.24 (95% c.i. 2.27 to 7.89) (compared with CFS 1–3) – AUC for CFS and 30-day mortality rate 0.74 (95% c.i. 0.69 to 0.78)
Vilches-Moraga *et al*. 2020^28^	Observational/cohort study	Prospective	ASA, reduced mobility, no POPS-GS	Adjusted outcomes	− 12-month mortality rate associated with CFS 5–9: adjusted HR 5.04 (95% c.i. 1.72 to 16.98) – 12-month readmission higher in frail patients (64% CFS ≥ 5 *versus* 31.7% CFS <5, *P* = 0.006)
Youssef *et al*. 2022^29^	Observational/cohort study	Retrospective	Age, ‘co-morbidities’, BMI	Adjusted outcomes	− Significant correlation with frailty and 30-day all-cause mortality rate CFS ≥4 adjusted OR 9.33 (95% c.i. 3.10 to 28.05) compared with CFS <4

POPS-GS, Perioperative Care of Older Persons-General Surgery; NELA-RPT, National Emergency Laparotomy Audit risk prediction tool; CFS, Clinical Frailty Scale; ROC, receiver operating characteristic; LOS, length of stay; AUC, area under the curve; NELA, National Emergency Laparotomy Audit; RPT, risk prediction tool; P-POSSUM, Portsmouth-Physiological and Operative Severity Score for the enUmeration of Mortality and Morbidity; RR, risk ratio; BMI, body mass index. OR/RR reported as adjusted unless only univariate analysis occurred.

### Secondary outcomes

The overall incidence of postoperative major complications was 24% (95% c.i. 2 to 83%, *I*^2^ = 67, *Fig. S1e*). Frailty was associated with an increase in postoperative major complications (*Fig. 3e*, *n* studies = 2, OR = 1.93, 95% c.i. 1.27 to 2.93, *P* = 0.002, *I*^2^ = 79%). Although frailty demonstrated a relationship with increased postoperative ICU admission, this was not significant (*Fig. 3f P* = 0.14). No relationship was demonstrated between frailty and unplanned reoperation (*Fig. 3g*, *P* = 0.73) or 30-day readmission (*Fig. 3h*, *P* = 0.90).

One study found that increasing individual frailty scores were associated with postoperative complications and increased duration of ICU stay, following adjustment for age and sex (*Table 3*)^26^. Three studies demonstrated increased duration in stay for patients with frailty. However, one of these studies only conducted univariate analysis and did not provide confidence intervals, and another found that following adjustment for sex and age the difference in duration of stay was no longer significant.^21,25,26^ Two studies further demonstrated no association with increasing individual frailty scores and 30-day readmission^25,26^.

### Rehabilitation and increased level of care on discharge

One study reported that increasing frailty scores were associated with an increased risk of requiring a higher level of care on discharge for older EL patients (*Table 3*)^20^. Similarly, another study reported that older adults with frailty admitted from home had an increased risk of not returning home^30^. Additionally, they found that these patients were twice as likely to require rehabilitation following EL^30^. Both studies adjusted for age and sex.

### CFS and other EL assessment tools

Two studies examined the addition of the CFS to pre-existing EL risk assessment tools (*Table 3*). One found that addition to the P-POSSUM and NELA Risk Prediction Tool (NELA-RPT) models improved the performance of both tools for older patients^24^. Conversely, another found that addition of the CFS to the NELA did not increase receiver operating characeristic values and that NELA outperformed the CFS for 30-day mortality rate^25^. One study found the CFS to be a weaker predictor of in-hospital, 30- and 90-day mortality rates when compared with sarcopenia (measured as reduced muscle mass on computed tomography)^22^.

## Discussion

This study has shown that frailty, defined using a CFS cut-off value of 4–6 (‘vulnerable’ to ‘moderately frail’) provides important prognostic information following EL. Frailty is significantly correlated with increased risk of short- and long-term mortality rates, as well as postoperative major complications and increased level of care on discharge. No relationship was demonstrated with unplanned reoperation, admission to ICU or 30-day readmission.

All included studies were published in the last 4 years, which likely correlates with recent NELA and WSES recommendations^2,3^. The pooled prevalence demonstrated that frailty according to the CFS was present in almost one-third of older EL patients. This was similar to the reported prevalence in the most recent NELA report (32.0% *versus* 34.7%)^29^. NELA reported increasing frailty with increasing age—49.6% of patients aged over 80 years had a CFS ≥ 5^31^. A similar relationship was demonstrated in two of the included studies that showed an approximately linear relationship with increasing age and individual frailty scores^25,28^. Consistent with included studies and prior research, age and sex were most frequently adjusted for and may be appropriate confounders to consider when using the CFS to measure frailty in the EL population^8^. Frailty was correlated with a significantly increased risk of death at all follow-up points, as well as major complications. Conversely, the relationship with frailty and 30-day readmission was insignificant. Mixed evidence regarding this relationship exists within the literature. Frailty using the CFS was associated with a four-fold increased risk of 30-day readmission in one Canadian study of emergency abdominal surgery patients, and a two-fold increased risk of readmission by 1 year in another study of critically ill older patients^32,33^.

There is a limited time for optimization of patients in the EL setting compared with elective surgery^10^. This may, in part, explain the historic lack of shared medical care of EL patients. The NELA and British Geriatrics Society recommended that patients 65 years and over and frail (defined by a CFS ≥ 5) should receive multidisciplinary input including early involvement of a geriatrician team^31,34^. In orthopaedics, an enhanced best practice tariff (BPT) including geriatrician input has been shown to significantly improve national UK postoperative outcomes for older hip fracture patients^35^. The NELA are working towards a similar BPT for the older frail EL patient^34^. Between 2016 and 2021, they reported the number of older patients with frailty assessed by geriatricians increased from 27.5% to 31.8%^31^. Of those in the over 65 years and frail group who underwent geriatrician review, 13.0% died in hospital, compared with 22.3% of those who did not receive geriatrician review. These proportions were similar for the frail and over 80 years age group. These numbers suggest significant benefits to routine care involving a coordinated multidisciplinary approach with geriatric liaison for the older patients with frailty from the beginning of the EL pathway^36,37^.

The implementation of tailored strategies by clinicians to evaluate risk before surgery and determine the type of care needed after surgery are essential for optimal stratification and care of this specific population^10^. NELA reported that frailty defined using the CFS is a crucial part of risk assessment, although there was mixed evidence regarding this addition to traditional tools such as P-POSSUM and NELA-RPT in this review^24,25^. They further stated that if frailty is present, a patient should be considered as ‘high risk’^31^. For high-risk patients, certain perioperative management techniques have the potential to reduce the physiological insult of EL. These may include timely surgery (less than 4 h), avoidance of prolonged use of opioid patient-controlled analgesias and regional anaesthesia^10^. Postoperative targeted bundles of care including supplemental nutrition, exercise interventions with early mobilization and deep breathing techniques, and early recognition and treatment of postoperative complications may further optimize outcomes for these patients^10^. Frailty was further identified as a risk factor for increased level of care on discharge in two included studies^20,30^. Tailored strategies implemented at an earlier stage of the EL pathway may help to return an older patient to their preoperative ‘baseline’, allowing them to return home. This may contribute to maintenance of independence and quality of life following EL.

There are recognized benefits and limitations to the CFS in the surgical setting. It has been validated for older surgical patients and may be determined before surgery. There is a limited time requirement and no formal training or additional equipment required. The assessment is straightforward to calculate and interpret by clinicians^7,8^. These features make the CFS an attractive tool for the EL setting. Conversely, the CFS may provide a baseline for a patient which may alter if the patient has a protracted illness before EL. In this setting of progressive illness, it can therefore prove challenging to measure. Furthermore, there is some evidence of variance in reporting due to subjective clinician-dependent assessments^3^. The clinician’s experience, combined with the ‘end-of-bed’ assessment—which may be affected by the patient’s acutely unwell presentation—may bias a clinician to over- or underestimate frailty^3^. However, good interrater reliability was demonstrated between clinicians in a large study of ICU patients across 129 centres^38^.

Frailty, as measured by the CFS, is significantly correlated with short- and long-term mortality rates following EL, as well as morbidity rates and increased level of care on discharge. The authors anticipate that frailty using the CFS will become an integral part of EL risk prediction over the coming years. Identifying frailty using the CFS may aid in patient-centred decision-making and implementation of tailored care strategies for these ‘high-risk’ patients, with the aim to reduce adverse outcomes following EL.

Several limitations were recognized in this review. Meta-analysis was limited to a certain extent due to variability in outcome reporting between studies. Some reported different outcomes by individual increasing frailty scores compared with 1 (‘very-fit’) or ‘1–3’ (‘very-fit’ to ‘managing well’). There was further variability between cut-off values to define frailty, although our meta-regression demonstrated that this variability was not significant in affecting the relationship with our primary outcome of 30-day mortality rates. A small number of studies were available for secondary endpoints which may reflect a power issue for non-significant outcomes, and significant heterogeneity existed for 6-month mortality rates and major complications. This may reduce the validity of some of our secondary outcomes.

## Supplementary Material

zrae078_Supplementary_Data

## Data Availability

Materials described in the manuscript are from published works and these papers are freely accessible.
