# Supplementary material for: Frailty using the Clinical Frailty Scale to predict short- and long-term adverse outcomes following emergency laparotomy: meta-analysis
Source: BJS Open. 2024 Aug 21;8(4):zrae078. doi: 10.1093/bjsopen/zrae078 (PMC11336663; doi:10.1093/bjsopen/zrae078)
Supplement: zrae078_Supplementary_Data [file zrae078_supplementary_data.zip › PROSPERO Amendment.docx]

Comment: An amendment to the PROSPERO protocol was submitted on 17 March 2024 to include the following:

Change to title – to **Frailty using the Clinical Frailty Scale predicts short- and long-term adverse outcomes following Emergency Laparotomy: A Systematic Review and Meta-Analysis**

Change to inclusion criteria – now to only include papers examining emergency laparotomy - rather than all emergency abdominal surgery

Change to methods – risk assessment tool changed to Quality in Prognosis Studies (QUIPS) tool

Change to methods - Frailty prevalence and overall incidence rates for all outcomes were reported as pooled prevalence and incidence (with associated 95% CI) using R version 3.6.3. Funnel plots of the incidence of 30-day mortality were used to screen for publication bias. Meta-regression was undertaken in R to examine the effect of individual increasing frailty scale numbers and 30-day mortality.

This amendment has been acknowledged but has not been uploaded to the PROSPERO online protocol yet.
