# Supplementary material for: Frailty using the Clinical Frailty Scale to predict short- and long-term adverse outcomes following emergency laparotomy: meta-analysis
Source: BJS Open. 2024 Aug 21;8(4):zrae078. doi: 10.1093/bjsopen/zrae078 (PMC11336663; doi:10.1093/bjsopen/zrae078)
Supplement: zrae078_Supplementary_Data [file zrae078_supplementary_data.zip › Supplementary tables and figures CFS final.docx]

**Supplementary Appendixes**

**Appendix S1.** Preferred Reporting Items for Systematic Reviews and Meta-Analyses (PRISMA) and Meta-analysis of Observational Studies in Epidemiology (MOOSE) checklists.

| **Section/topic** | **#** | **Checklist item** | **Reported on page #** |
| --- | --- | --- | --- |
| **TITLE** | | |  |
| Title | 1 | Identify the report as a systematic review, meta-analysis, or both. | 1 (Title Page) |
| **ABSTRACT** | | |  |
| Structured summary | 2 | Provide a structured summary including, as applicable: background; objectives; data sources; study eligibility criteria, participants, and interventions; study appraisal and synthesis methods; results; limitations; conclusions and implications of key findings; systematic review registration number. | 2 (Abstract page) |
| **INTRODUCTION** | | |  |
| Rationale | 3 | Describe the rationale for the review in the context of what is already known. | 3 |
| Objectives | 4 | Provide an explicit statement of questions being addressed with reference to participants, interventions, comparisons, outcomes, and study design (PICOS). | 3 |
| **METHODS** | | |  |
| Protocol and registration | 5 | Indicate if a review protocol exists, if and where it can be accessed (e.g., Web address), and, if available, provide registration information including registration number. | 5 |
| Eligibility criteria | 6 | Specify study characteristics (e.g., PICOS, length of follow-up) and report characteristics (e.g., years considered, language, publication status) used as criteria for eligibility, giving rationale. | 5 |
| Information sources | 7 | Describe all information sources (e.g., databases with dates of coverage, contact with study authors to identify additional studies) in the search and date last searched. | 5 |
| Search | 8 | Present full electronic search strategy for at least one database, including any limits used, such that it could be repeated. | 5, Appendix S2 |
| Study selection | 9 | State the process for selecting studies (i.e., screening, eligibility, included in systematic review, and, if applicable, included in the meta-analysis). | 6 |
| Data collection process | 10 | Describe method of data extraction from reports (e.g., piloted forms, independently, in duplicate) and any processes for obtaining and confirming data from investigators. | 6 |
| Data items | 11 | List and define all variables for which data were sought (e.g., PICOS, funding sources) and any assumptions and simplifications made. | 6 |
| Risk of bias in individual studies | 12 | Describe methods used for assessing risk of bias of individual studies (including specification of whether this was done at the study or outcome level), and how this information is to be used in any data synthesis. | 7 |
| Summary measures | 13 | State the principal summary measures (e.g., risk ratio, difference in means). | 8 |
| Synthesis of results | 14 | Describe the methods of handling data and combining results of studies, if done, including measures of consistency (e.g., I^2^) for each meta-analysis. | 8 |
| **Section/topic** | **#** | **Checklist item** | **Reported on page #** |
| Risk of bias across studies | 15 | Specify any assessment of risk of bias that may affect the cumulative evidence (e.g., publication bias, selective reporting within studies). | 8 |
| Additional analyses | 16 | Describe methods of additional analyses (e.g., sensitivity or subgroup analyses, meta-regression), if done, indicating which were pre-specified. | 8 |
| **RESULTS** | | |  |
| Study selection | 17 | Give numbers of studies screened, assessed for eligibility, and included in the review, with reasons for exclusions at each stage, ideally with a flow diagram. | **9** |
| Study characteristics | 18 | For each study, present characteristics for which data were extracted (e.g., study size, PICOS, follow-up period) and provide the citations. | 9 |
| Risk of bias within studies | 19 | Present data on risk of bias of each study and, if available, any outcome level assessment (see item 12). | 9 |
| Results of individual studies | 20 | For all outcomes considered (benefits or harms), present, for each study: (a) simple summary data for each intervention group (b) effect estimates and confidence intervals, ideally with a forest plot. | 10 |
| Synthesis of results | 21 | Present results of each meta-analysis done, including confidence intervals and measures of consistency. | 10 |
| Risk of bias across studies | 22 | Present results of any assessment of risk of bias across studies (see Item 15). | 9 |
| Additional analysis | 23 | Give results of additional analyses, if done (e.g., sensitivity or subgroup analyses, meta-regression [see Item 16]). | 10 |
| **DISCUSSION** | | |  |
| Summary of evidence | 24 | Summarize the main findings including the strength of evidence for each main outcome; consider their relevance to key groups (e.g., healthcare providers, users, and policy makers). | 12 |
| Limitations | 25 | Discuss limitations at study and outcome level (e.g., risk of bias), and at review-level (e.g., incomplete retrieval of identified research, reporting bias). | 12 |
| Conclusions | 26 | Provide a general interpretation of the results in the context of other evidence, and implications for future research. | 12 |
| **FUNDING** | | |  |
| Funding | 27 | Describe sources of funding for the systematic review and other support (e.g., supply of data); role of funders for the systematic review. | 14 |

| **Item No** | **Recommendation** | **Reported on Page No** |
| --- | --- | --- |
| Reporting of background should include | | |
| 1 | Problem definition | 3 |
| 2 | Hypothesis statement | 3 |
| 3 | Description of study outcome(s) | 3 |
| 4 | Type of exposure or intervention used | 3 |
| 5 | Type of study designs used | 5 |
| 6 | Study population | 5 |
| Reporting of search strategy should include | | |
| 7 | Qualifications of searchers (eg, librarians and investigators) | 5 |
| 8 | Search strategy, including time period included in the synthesis and key words | Appendix S2 |
| 9 | Effort to include all available studies, including contact with authors | 6 |
| 10 | Databases and registries searched | 6 |
| 11 | Search software used, name and version, including special features used (eg, explosion) | 6 |
| 12 | Use of hand searching (eg, reference lists of obtained articles) | 7 |
| 13 | List of citations located and those excluded, including justification | 7 |
| 14 | Method of addressing articles published in languages other than English | 7 |
| 15 | Method of handling abstracts and unpublished studies | 7 |
| 16 | Description of any contact with authors | 7 |
| Reporting of methods should include | | |
| 17 | Description of relevance or appropriateness of studies assembled for assessing the hypothesis to be tested | 8 |
| 18 | Rationale for the selection and coding of data (eg, sound clinical principles or convenience) | 8 |
| 19 | Documentation of how data were classified and coded (eg, multiple raters, blinding and interrater reliability) | 8 |
| 20 | Assessment of confounding (eg, comparability of cases and controls in studies where appropriate) | 8 |
| 21 | Assessment of study quality, including blinding of quality assessors, stratification or regression on possible predictors of study results | 8 |
| 22 | Assessment of heterogeneity | 9 |
| 23 | Description of statistical methods (eg, complete description of fixed or random effects models, justification of whether the chosen models account for predictors of study results, dose-response models, or cumulative meta-analysis) in sufficient detail to be replicated | 9 |
| 24 | Provision of appropriate tables and graphics | Attached Figures |
| Reporting of results should include | | |
| 25 | Graphic summarizing individual study estimates and overall estimate | Attached Figures |
| 26 | Table giving descriptive information for each study included | Attached Tables |
| 27 | Results of sensitivity testing (e.g, subgroup analysis) | Attached Figures |
| 28 | Indication of statistical uncertainty of findings | 10 |

From: Stroup DF, Berlin JA, Morton SC, et al, for the Meta-analysis Of Observational Studies in Epidemiology (MOOSE) Group. Meta-analysis of Observational Studies in Epidemiology. A Proposal for Reporting. JAMA. 2000;283(15):2008-2012. doi: 10.1001/jama.283.15.2008.

# **Appendix S2.** Search string exemplar applied to the MEDLINE (OVID) electronic database

The search was conducted on 05/10/23 and then re-run on 01/02/24

**Ovid MEDLINE(R) Epub Ahead of Print, In Process & Other Non-Indexed Citations, Ovid MEDLINE (R) Daily, and Ovid MEDLINE (R) 1946-Present:**

1 exp frail OR ‘CFS’ OR ‘Clinical Frailty Scale’

2 (clin* OR functional OR physical) adj3 (frail* OR measure* OR predictor* OR test*)mp

3 1 OR 2

4 exp laparotomy

5 exp Digestive system surgical procedures

6 exp colorectal surgery/ OR exp general surgery/ OR exp emergency laparotomy/ OR exp acute laparotomy

7 (abdom* surgery OR abdom* operation OR laparotomy).mp

8 ((gastrointestinal OR GI OR colorectal OR digestive system(and surg*)).mp

9 4 OR 5 OR 6 OR 7 OR 8

10 3 AND 9

11 limit 13 to (English language and adults)

mp=title, abstract, original title, name of substance word, subject heading word, floating sub-heading word, keyword heading word, organism supplementary concept word, protocol supplementary concept word, rare disease supplementary concept word, unique identifier, synonyms.

adj = adjacency.

exp=explode

**Table S1**. Quality assessment of included studies using the QUality In Prognosis Studies (QUIPS) tool

| **Study** | **Bias domain** | | | | | |
| --- | --- | --- | --- | --- | --- | --- |
|  | **Study participation** | **Study attrition** | **Prognostic factor measurement** | **Outcome measurement** | **Study confounding** | **Statistical analysis and reporting** |
| Alder ’21 | Low | Low | Low | Low | Low | Low |
| Carter ’20 | Low | Low | Low | Low | Low | Low |
| Ethiraj ’22 | Low | Moderate | Low | Low | Moderate | High |
| Isand ’23 | Low | Low | Low | Low | Low | Low |
| Palaniappan ’22 | Moderate | Moderate | Low | Low | Low | Low |
| Parmar ’21 | Low | Low | Low | Low | Low | Low |
| Ramsay ’22 | Moderate | Moderate | Low | Low | Low | Low |
| Vilches-Moraga ’20 | Low | Low | Low | Low | Low | Low |
| Youssef ’22 | Low | Low | Low | Low | Low | Low |
| Park ’24 | Low | Low | Low | Low | Low | Low |
| Mak ’24 | Low | Low | Low | Low | Moderate | Low |
| Hajibandeh ’24 | Low | Low | Low | Low | Low | Low |

**Table S2.** Patient demographic characteristics in each included study

| **Study** | **Total Sample size** | **Age in  years (f/nf), mean** [**±**](https://www.bing.com/ck/a?!&&p=168085235617ed76f22919b5cabb8cccd88c216b913f3163020463b1f60ff1e3JmltdHM9MTY1ODE0NzEyOSZpZ3VpZD0wYmE3OWQ4Yi03ZWRkLTQ2MmQtOGI0My1mYmI0YmNhMGEzMjYmaW5zaWQ9NTE4NQ&ptn=3&fclid=b5c92a6d-0694-11ed-8dd1-4e38d69fa304&u=a1aHR0cHM6Ly9ob3d0b3R5cGVhbnl0aGluZy5jb20vcGx1cy1taW51cy1zeW1ib2wv&ntb=1) **SD** | **Gender M, F (f/ nf)** | **ASA 1-2 (f/ nf)** | **ASA 3-5**  **(f/ nf)** |
| --- | --- | --- | --- | --- | --- |
| Alder ‘21 | 153 | 79.0 ± 6.7 | 57, 96 | 55 | 98 |
| Carter ‘20/Parmar ‘21 | 934 | 65-75 = 424, 75-85 = 386, 85+ = 124 | 396, 538 | 314 | 618 |
| Ethiraj ‘22 | 110 | 71.2 ± 9.3 | 82, 28 | - | - |
| Hajibandeh ’24 | 1043 | 62.0 ±1.2 | 514, 529 | 456 | 587 |
| Isand ‘23 | 793 | 63.1 ± 17.5 | 392, 401 | 465 | 328 |
| Palaniappan ‘22/ Ramsay ‘22 | 2246 (812 missing a CFS) | 65.0 ±17.8 | 1099, 1147 | 916 | 1, 330 |
| Vilches-Moraga ‘20 | 113 | 81.9 ± 4.7 | 53, 60 | 49 | 64 |
| Youssef ‘22 | 191 | 75.6 ± 2.5 | 106, 95 | - | - |
| Park ’24 | 629 | 80.0 ± 10.33/ 74.0 ± 10.54 | 34, 58/ 252, 285 | 22/168 | 68/358 |
| Mak ‘24 | 306 | 65.5±23.7 | 146, 160 | 155 | 141 |

**Figure S1:**

**Overall Incidence of Primary and Secondary Outcomes:**

- A. 30-Day Mortality:


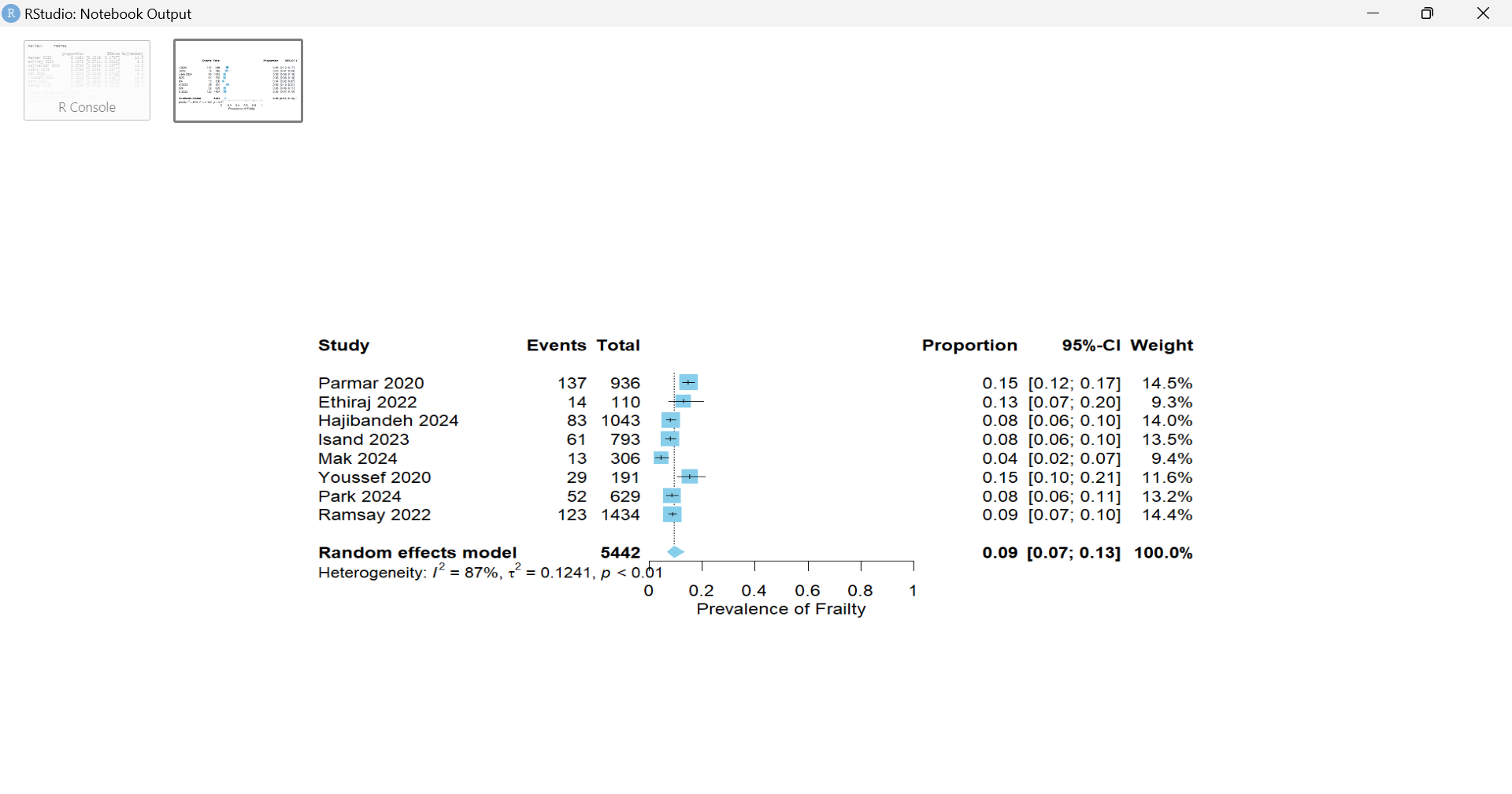


- B. 90-Day Mortality:


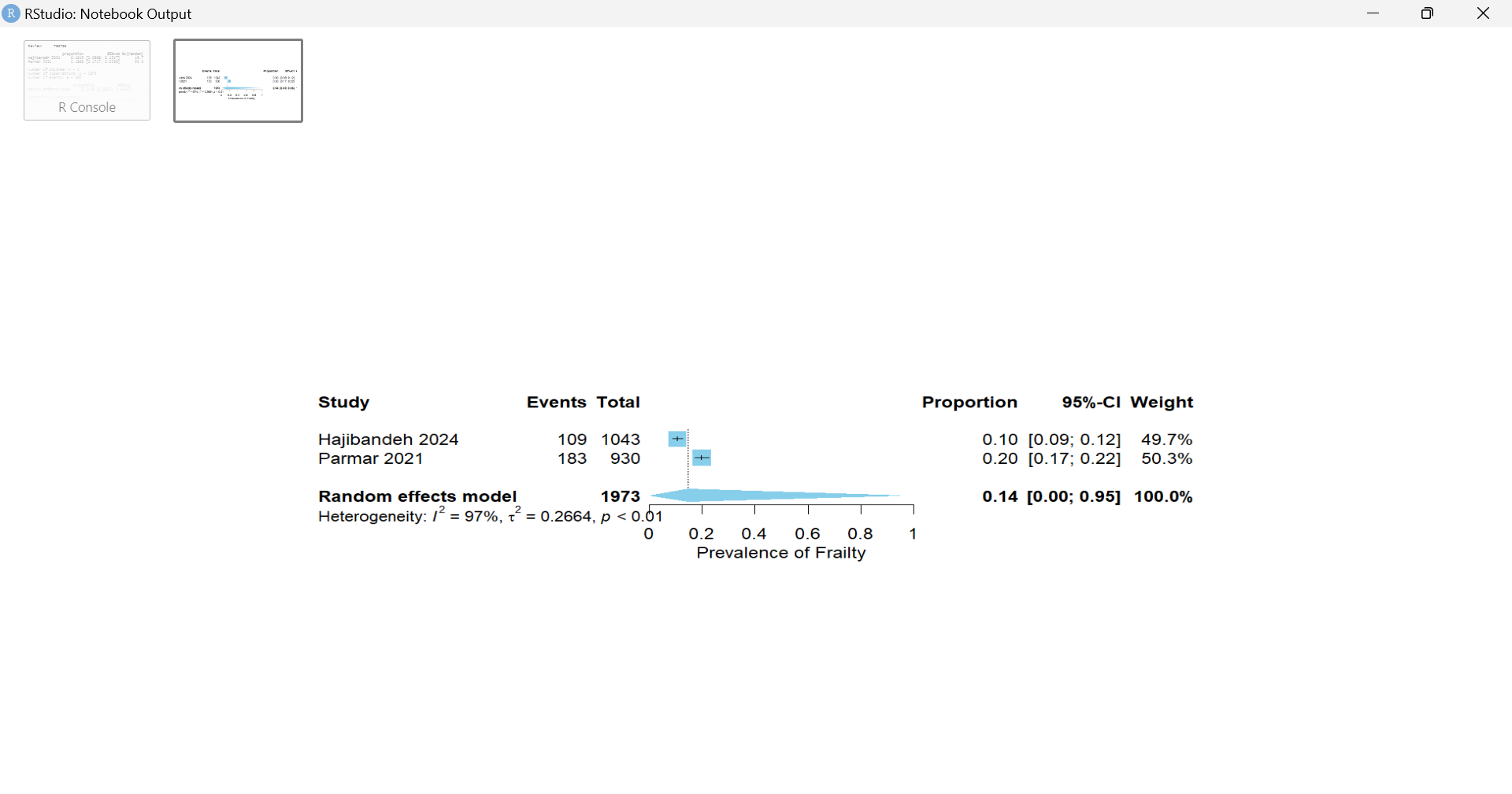


- C. 6-Month Mortality:


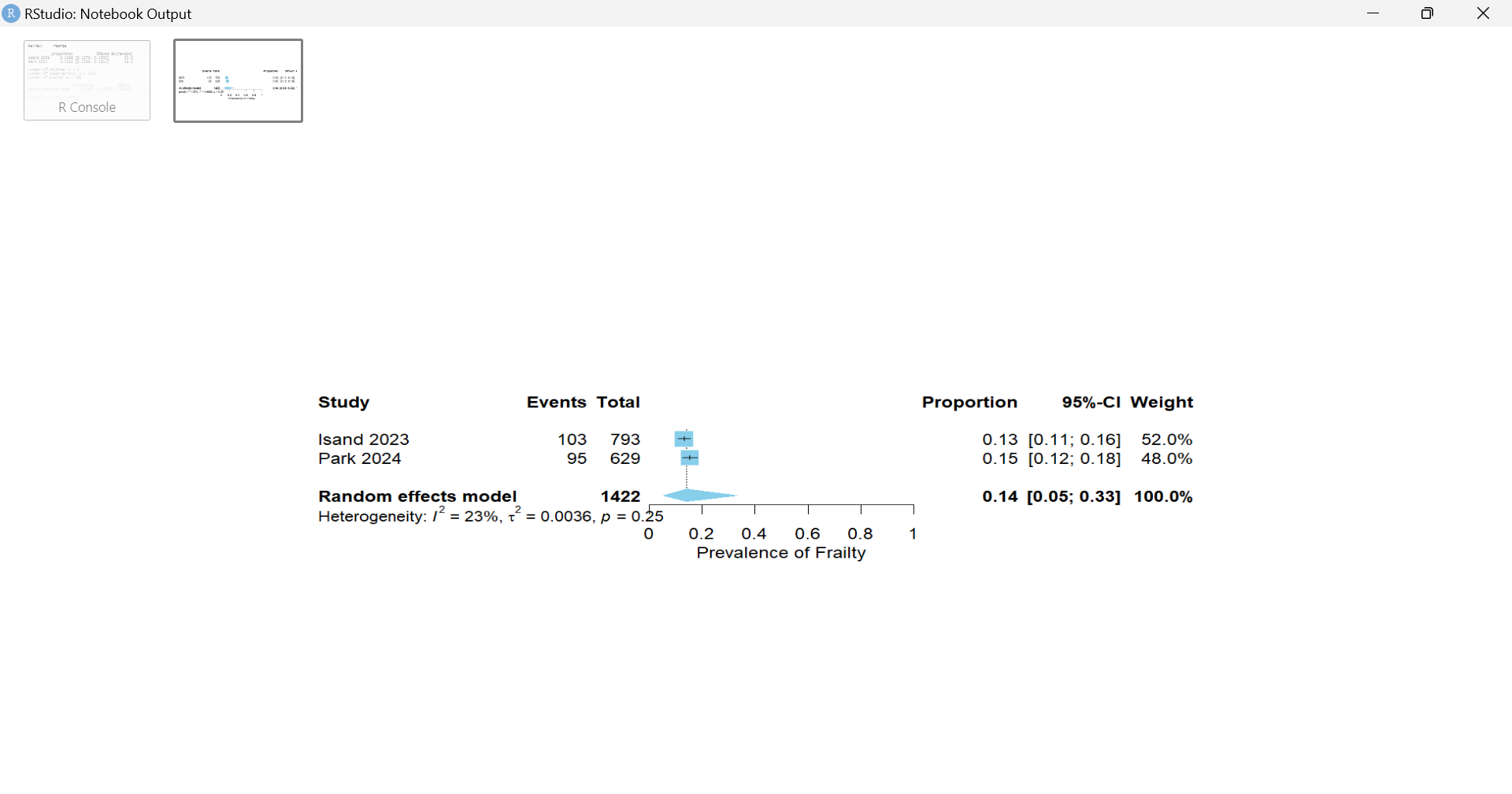


- D. One-Year Mortality:


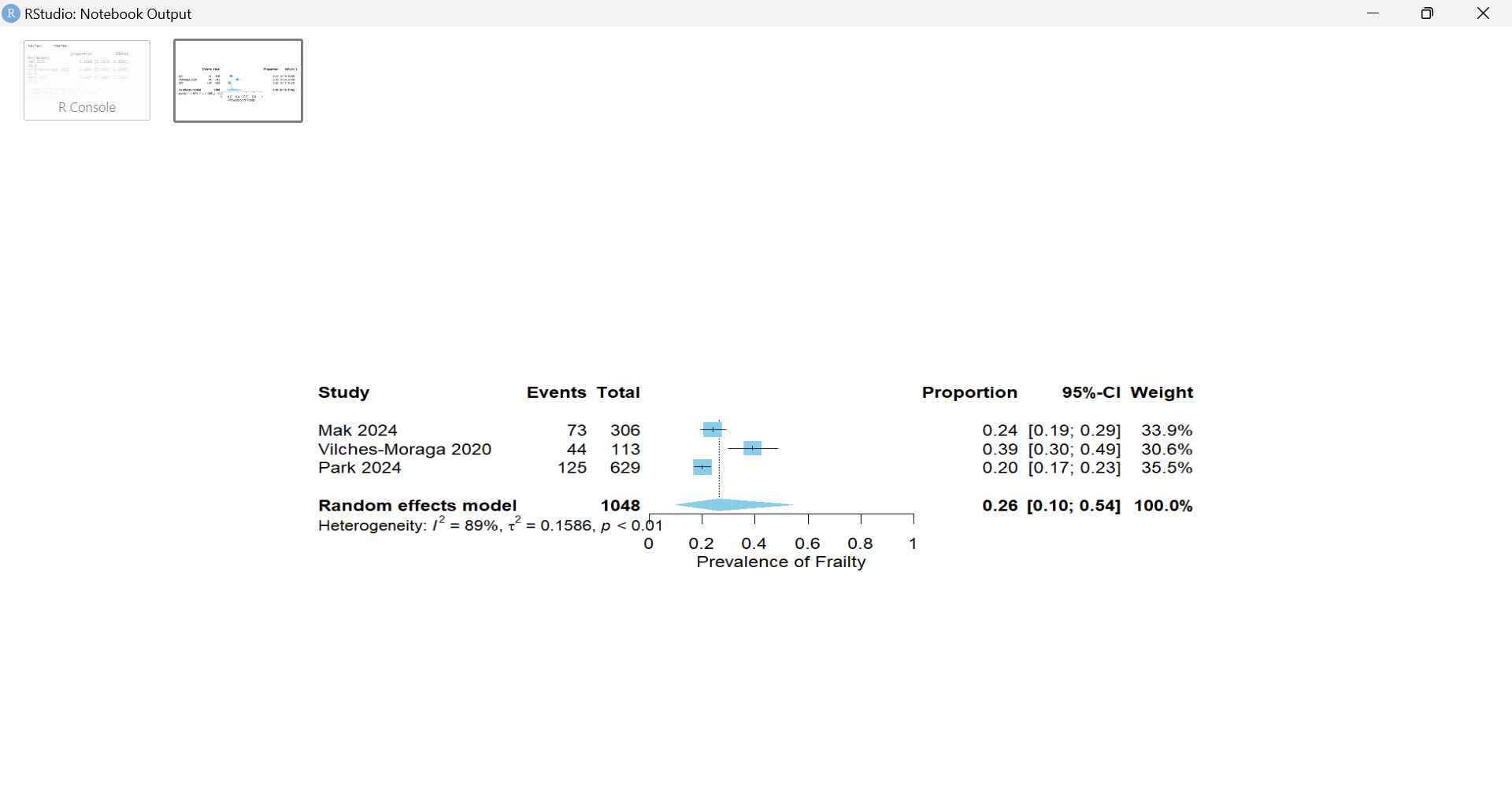


- E. Major Complications:


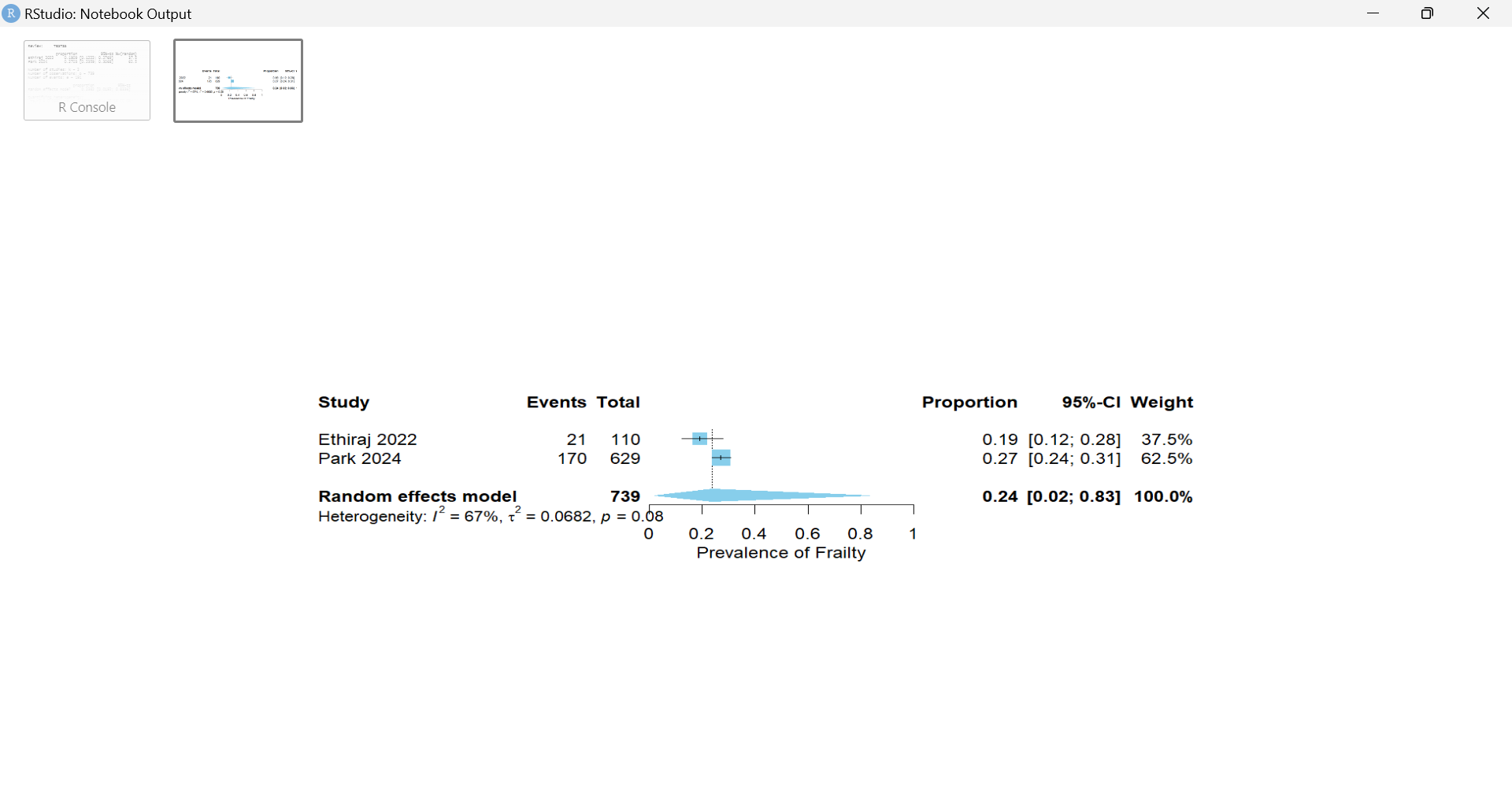


- F. ICU Admission:


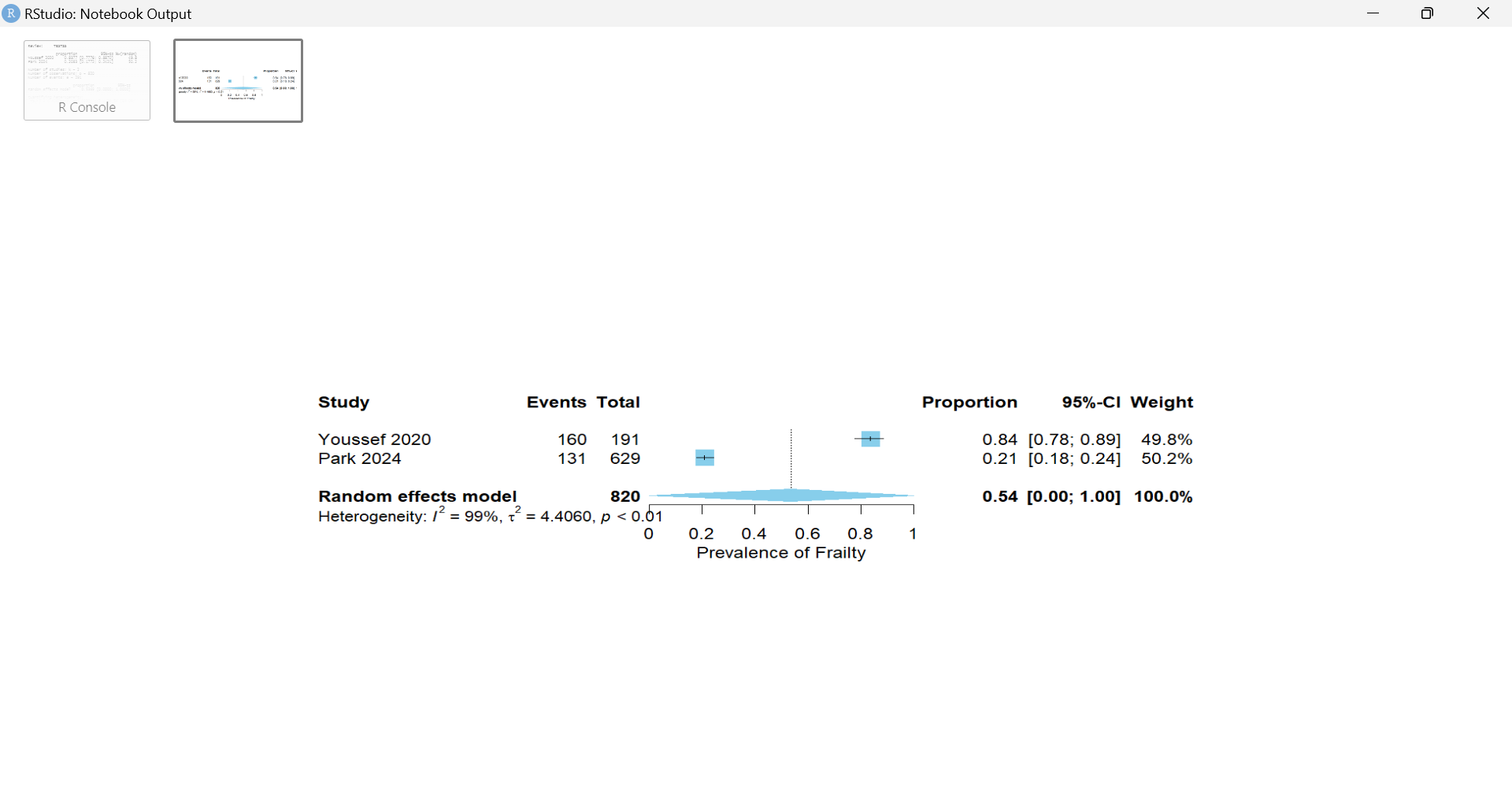


- G. Unplanned Operations:


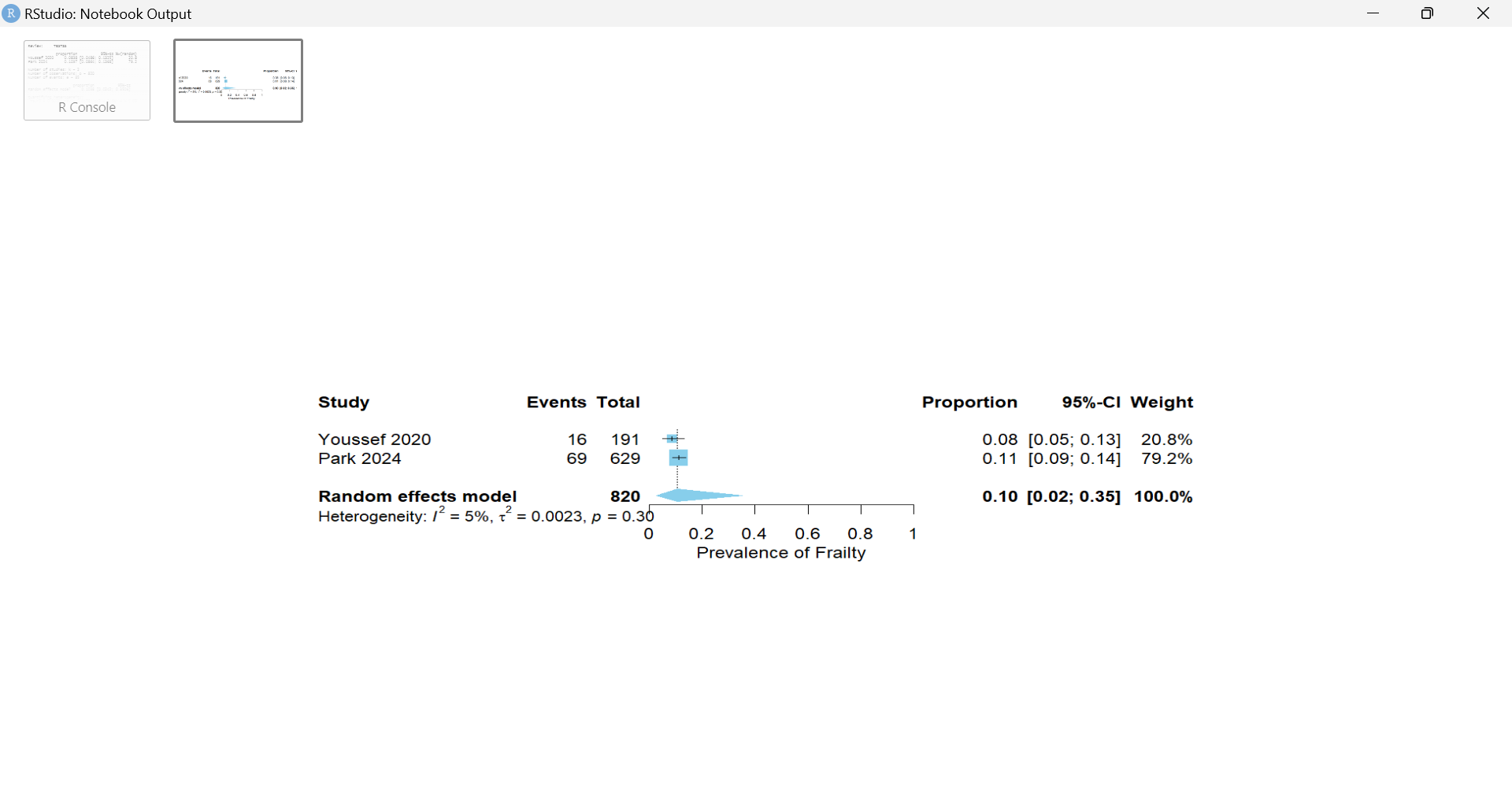


- H. 30-Day Readmission


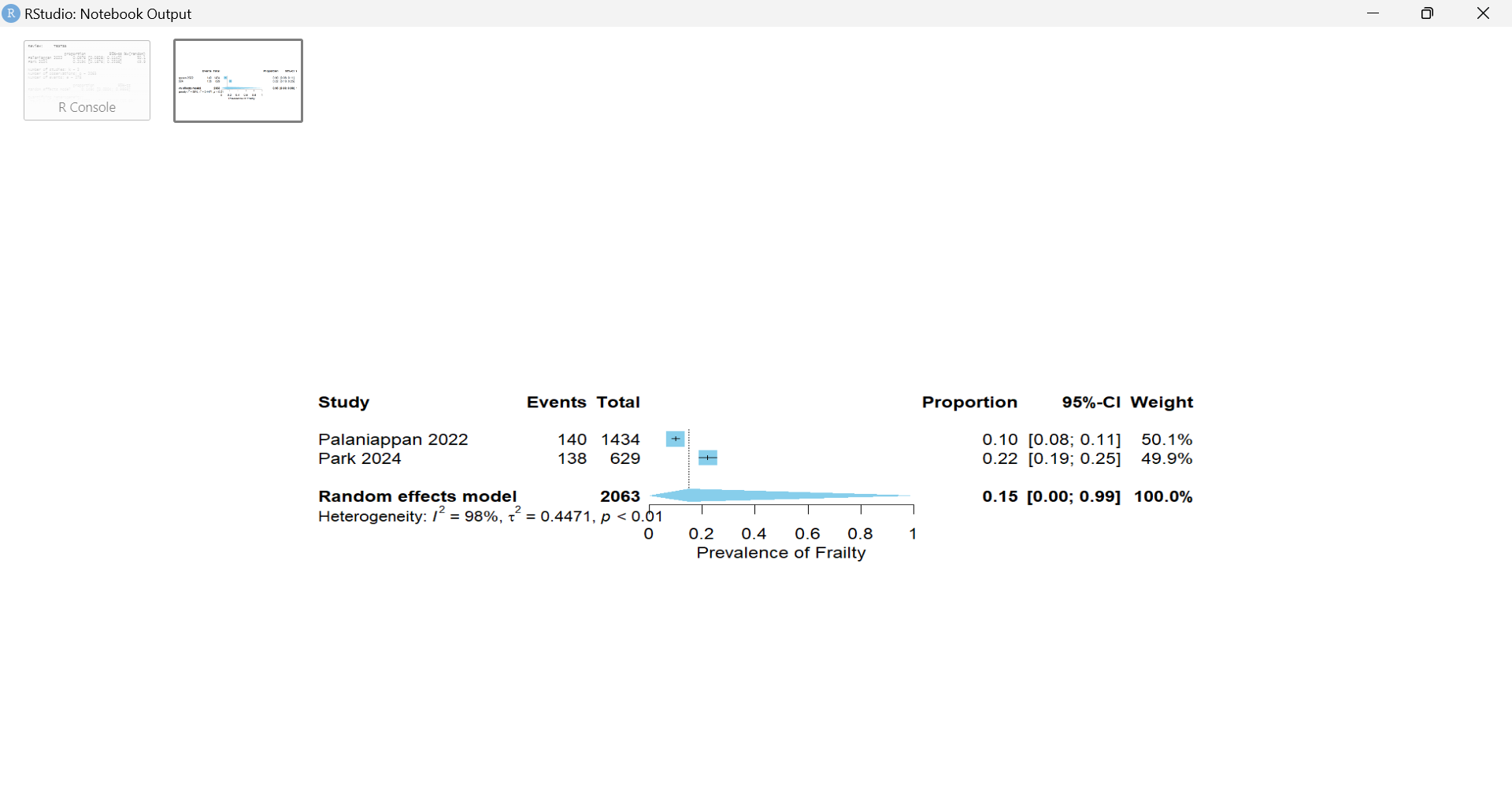


**Figure S2: Subgroup Analysis – Over 55 30-Day Mortality**

**
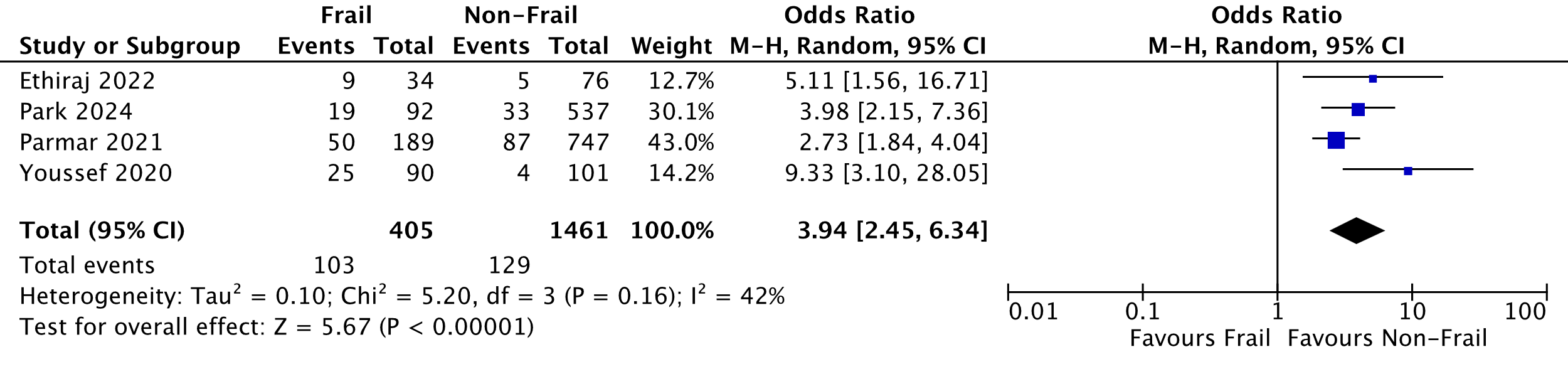
**
